# Naturally Occurring Plant Food Toxicants and the Role of Food Processing Methods in Their Detoxification

**DOI:** 10.1155/2023/9947841

**Published:** 2023-04-27

**Authors:** Markos Makiso Urugo, Tadele Tuba Tringo

**Affiliations:** ^1^Department of Food Science and Postharvest Technology, College of Agricultural Sciences, Wachemo University, Hosanna, Ethiopia; ^2^College of Engineering and Agro-Processing Technology, Arba Minch University, Arba Minch, Ethiopia

## Abstract

Some plant foods evolve defense mechanisms to protect themselves from predators by producing inherent chemicals as secondary metabolites such as cyanogenic glycosides, glycoalkaloids, glucosinolates, pyrrolizidine alkaloids, and lectins. These metabolites are beneficial for the plant itself but toxic to other organisms, including human beings. Some of these toxic chemicals are believed to have therapeutic benefits and are therefore used to protect against chronic health complications such as cancer. Inversely, short- and long-term exposure to significant amounts of these phytotoxins may end up with chronic irreversible negative health problems in important organ systems, and in severe cases, they can be carcinogenic and fatal. A systematic literature search of relevant published articles indexed in Google Scholar®, PubMed®, Scopus®, Springer Link®, Web of Science®, MDPI®, and ScienceDirect databases was used to obtain the necessary information. Various traditional and emerging food-processing techniques have been found to considerably reduce most of the toxicants in the food to their safest level. Despite their ability to preserve the nutritional value of processed foods, emerging food processing methods have limited application and accessibility in middle- and low-income countries. As a consequence, much more work is recommended on the implementation of emerging technologies, with additional scientific work on food processing methods that are effective against these naturally occurring plant food toxicants, particularly pyrrolizidine alkaloids.

## 1. Introduction

Food is one of every living entity's basic necessities, and it is composed of different nutrients that can be consumed by animals, including humans, for sustenance [1, 2]. Almost all foods are made from plants or animals, and many plants or plant components are used as food. According to the Royal Botanic Gardens, Kew, more than 7000 species of vascular plants are edible, used for human consumption, and cultivated for food [3]. Food crops produce a wide range of non-nutrient secondary metabolites in addition to nutrients. These secondary metabolites have chemical structures ranging from simple organic compounds to sophisticated molecules like proteins [4, 5]. Among the plant metabolites, inherent plant toxicants are believed to play an ecological role in plant physiology, proliferation, or defense. Also, some of the secondary metabolites repel predators and are thus toxic or unpleasant to humans [5, 6], while others have different purposes, including plant physiological defense against insects, bacteria, fungi, and viruses [4, 5, 7, 8].

Inherent plant toxins are not harmful to the organism itself, but they are dangerous to other species, including humans, when consumed [5]. The structures of these chemicals differ, as do their biological functions and toxicity [7, 9]. Plants' secondary metabolites can have a negative impact on consumers' health, ranging from acute to chronic toxicity [9]. Acute toxicity includes nausea, dizziness, stomach discomfort, vomiting, and skin allergies, whereas chronic health consequences can cause irreversible harm to important organ systems such as the immune system, kidneys, and reproductive system, and in severe cases, they can be carcinogenic and fatal [10, 11].

The presence of natural toxins in food and feed is undeniably a significant food safety problem for both scientists and regulatory bodies [4]. Importantly, it is believed that dietary exposure to these naturally occurring nonnutrient compounds might far outweigh exposure to any sort of man-made chemical found in food [5, 12, 13]. Taking consumer health into account, WHO collaborated with the FAO of the United Nations to develop the Joint Expert Committee on Food Additives (JECFA), responsible for assessing health risks posed by foods containing natural toxins. Based on JECFA evaluations, Codex Alimentarius established international rules and guidelines to limit the amount of natural poisons in food [14]. The EU Council Directive on Flavorings (88:388:EU) regulates intrinsic toxicants from many plant source materials, including plant foods, herbs, and spices, such as coumarin, thujone, safrole, and hydrocyanic acid (originating from cyanogenic glycosides) [6].

Food safety is recognized as an essential component of food security, thus guaranteeing food safety is vital [15]. Food safety laws in developing countries are weak, which allows individuals to be constantly exposed to considerable amounts of intrinsic food toxicants [16–18]. According to the literature, various traditional and emerging food-processing techniques such as drying [19], boiling/cooking [20], fermentation [21], germination [22], microwave heating [23], and high-pressure processing (HPP) [24] are reported as effective mitigation strategies to detoxify toxicants. As a result, this review article summarizes natural food toxicants of plant sources and the role of traditional and novel food processing techniques in their detoxification.

## 2. Methodology

A systematic literature search of relevant published articles indexed in Google Scholar®, PubMed, Scopus®, Springer Link, Web of Science®, MDPI, and ScienceDirect databases using the following keywords: “Plant food toxicants,” “inherent plant food toxicants,” “secondary metabolites of plant food,” “traditional food processing methods to detoxify inherent plant food toxins,” and “novel food processing methods used to detoxify inherent toxic compounds of plant food” were used. Furthermore, each selected publication's books and references were examined to obtain additional information on naturally occurring plant food toxicants and the food processing methods used to detoxify the toxicants. There are several other phytotoxins such as tannins, phytoestrogens, phytates, and biogenic amines; however, the authors decided to focus on cyanogenic glycosides, glycoalkaloids, glucosinolates, pyrrolizidine alkaloids, and lectins. This is because these five plant food toxicants are commonly encountered and found in the major staple foods consumed by a significant proportion of the world's population and are more potent when compared to others.

## 3. Naturally Occurring Plant Food Toxicants

Several plant species grown for food produce a large number of phytotoxins that are different from the primary metabolic intermediates and products [4]. Some of the inherent toxicants with adverse health consequences produced as secondary metabolites by different plant foods are summarized below.

### 3.1. Cyanogenic Glycosides

**Figure 1:**
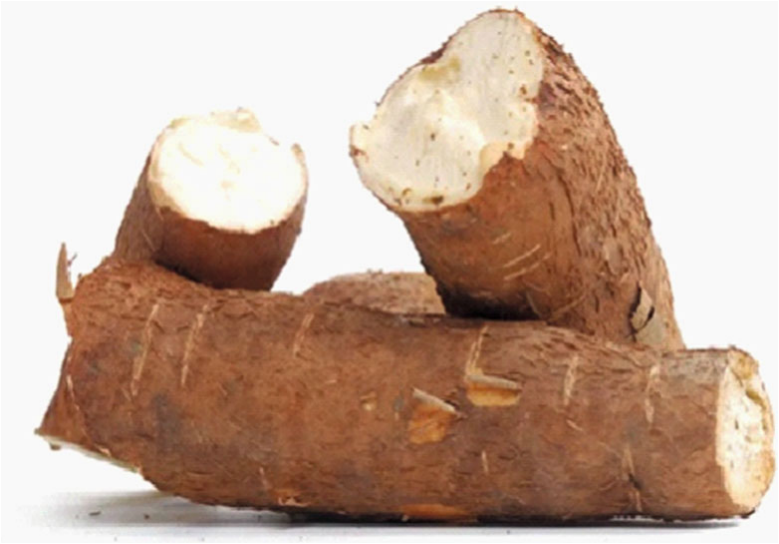
Cassava root [34].

Cyanogenic glycosides are amino acid-derived plant constituents produced as secondary metabolites and used as a defense mechanism against a variety of predators [19, 25–27]. They produce hydrogen cyanide (HCN) and are found in the members of the *Compositae*, *Fabaceae*, *Leguminosae*, *Linaceae*, and *Rosaceae* families [19, 26]. Particularly they occur in cassava (*Manihot esculenta* Crantz) (linamarin, 900–2000 mg HCN/kg dry matter), [28], flaxseed (*Linum usitatissimum*) (264–354 mg/kg) [29], sorghum (*Sorghum bicolor* L.) (dhurrin, 30% dry weight) [30], apricot fruits (*Prunus armeniaca*) (49–4000 mg/kg) [31], apple (*Malus domestica*) (amygdalin, 1-4 mg/g) [25], cocoyam (*Colocasia esculenta*) (21.0–171.3 mg/kg dry matter) [32], and bamboo (taxiphyllin, 1000–8000 mg HCN/kg) [27, 33].  Figure 1 indicates cassava root, which is known to contain a high amount of cyanogenic glycoside.

#### 3.1.1. Structure

**Figure 2:**
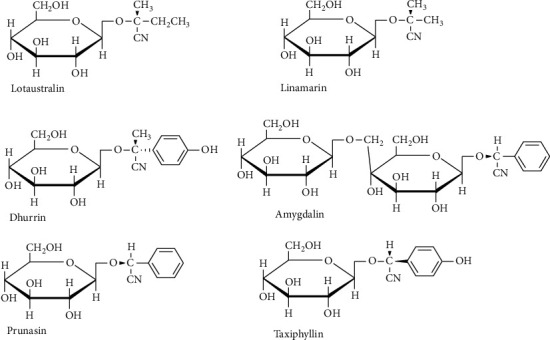
Structure of cyanogenic glycosides in major edible plants [19].

Approximately 25 cyanogenic glycosides are predominantly found in the edible section of plants [35]. Cyanogenic glycosides are derived from the amino acids leucine, isoleucine, tyrosine, phenylalanine, and valine and the non-proteinogenic amino acid, cyclopentenylglycine [19, 26]. Linamarin and lotaustralin are synthesized from the amino acids leucine, isoleucine, and valine. Dhurrin is formed from tyrosine, whereas amygdalin and prunasin are derived from phenylalanine [36].  Figure 2 depicts the major cyanogenic glycosides of different plant foods.

#### 3.1.2. Toxicity

The ability of cyanogenic glycosides to release HCN determines their toxicity [19, 20, 35]. Dietary cyanide exposure can cause acute poisoning and has been implicated in the etiology of various chronic disorders [37, 38]. The interaction of cyanogenic glycosides with the hydrolytic glucosidase enzyme is required for toxicity. Following that, the glycoside degrades to a sugar and a cyanohydrin, which quickly decomposes to hydrogen cyanide and an aldehyde or ketone [28]. Amygdalin, a cyanogenic glycoside found in bitter almonds and peach stones, is transformed into glucose, benzaldehyde, and poisonous hydrogen cyanide [19, 27, 39].

Consumption of underprocessed cassava has been linked to cyanide poisoning, tropical ataxic neuropathy (TAN) disease, and Konzo [19, 40, 41]. Cyanides are among the most potent toxins and come in a variety of forms. HCN and cyanide salts such as potassium cyanide, sodium cyanide, and calcium cyanide are the most prevalent, as cyanide salts can react with acids to produce HCN [19, 20, 37, 42]. Cyanide inhibits cytochrome oxidase, which prevents oxygen use and results in cytotoxic anoxia. This reduces the use of oxygen in the tissues. Moreover, as the blood glucose and lactic acid levels rise, the ATP/ADP ratio falls, indicating a shift from aerobic to anaerobic metabolism [43, 44]. Furthermore, the cyanide ion (CN^−^) has a high affinity for the trivalent iron (Fe^3+^) of cytochrome oxidase in humans and is easily absorbed through the digestive and respiratory tracts [19, 45].

Acute cyanide intoxication primarily affects the central nervous system (CNS), as well as the respiratory, cardiovascular, and endocrine systems. In particular, it can cause fast breathing, a reduction in blood pressure, dizziness, headache, stomachaches, vomiting, diarrhea, cyanosis with twitching and convulsions, mental confusion, and mortality [19, 46]. The fatal doses described in the literature greatly vary. However, the mean lethal dose of cyanide by mouth in human adults is estimated to be in the range of 50 to 200 mg, and death is rarely delayed for more than 1 hour if untreated [46].

Chronic cyanide exposure is known to cause symptoms that differ from those observed in acute doses. It has been linked to a number of health problems, particularly among cassava-eating people [20, 35]. Chronic cyanide toxicity has been linked to health issues such as malnutrition, congenital abnormalities, neurological problems, and myelopathy. Goiter or thyroid gland swelling has also been reported in populations where cyanogenic glycoside levels in cassava diets exceed 10–50 mg/kg [19, 20, 35].

#### 3.1.3. Cyanogenic Glycoside Detoxification Methods

Cyanide levels in edible plants are greatly lowered through processing to an acceptable Food and Agriculture Organization (FAO)/World Health Organization (WHO) limit of 10 mg HCN/kg dry weight [47]. Extensive scientific research has been conducted on cyanogenic glycoside mitigation techniques. They are summarized below.

*(1) Peeling*. Peeling is the first stage in the processing of cassava roots. Cassava peel has a higher concentration of cyanide than cassava pulp [48]. By peeling the root, 50% of the cyanogenic glycosides in the root can be eliminated. The peel of bitter cassava contains 650 ppm of total cyanide, and the pulp comprises 310 ppm. On the other hand, the sweet cassava variety cyanide concentration in peel and pulp was determined to be 200 ppm and 38 ppm, respectively [49]. In the sweet varieties where the peel has been removed, the pulp can be consumed immediately after boiling, while bitter varieties require additional detoxification measures before eating [42].

*(2) Grating*. Grating is a size reduction process, and therefore it increases the surface area that allows for greater contact of linamarin and linamarase for an easier detoxification process [19, 42]. The smaller size permits intracellular linamarin to be liberated from the cell and react with the external linamarase enzyme to create volatile HCN [42, 50]. The cyanide content in grated cassava roots is determined by the period of contact between the glucoside and glucosidase in aqueous media. Although grating alone is insufficient for detoxification, it can be used with other procedures to improve HCN evaporation or reduction via fermentation [42, 51]. It has also been found that grated-mash fermentation reduced the cyanogenic glycoside concentration in tofu, a typical fermented cassava product, by 85.5% in 72 hours [52].

*(3) Drying*. Drying is one of the most effective methods for removing cyanogenic glycosides from plant foods [19, 20]. There are several drying methods available to reduce cyanogen content in food products, including sun, oven, freeze, and superheated steam [43, 53]. On the other hand, superheated steam drying at 120–160°C resulted in a considerable breakdown of taxiphyllin, which induces bitterness in bamboo shoots [53], whereas oven-drying at 60°C after grinding for 8 hours resulted in a 95% reduction in cyanogen content [54].

*(4) Boiling/Cooking*. Cooking and boiling are among the most efficient procedures for reducing cyanogenic chemicals in plant foods [20, 53]. These activities appear to enhance cell wall rupture, allowing for the movement of cell contents such as antinutrients and hazardous chemicals [55]. An early report on the effect of boiling on the cyanogenic glycoside content of cassava revealed that boiling for 25 minutes decreased bound cyanogenic glycoside by 45 to 50% [56]. However, other studies revealed that boiling considerably lowered the cyanogenic glycoside content of cassava. Boiling for 10 minutes reduced the cyanogenic glycoside in the shoots of *Bambusa vulgaris* by 67.84–76.92%, and further boiling of the shoots for 10 minutes reduced the cyanogen concentration by up to 87% [53]. Furthermore, steaming was shown to greatly lower the cyanide concentration of cassava flour (raw material) (72.6%), and the cyanide content further dropped following saccharification and fermentation, eventually decreasing by 81.5% [57].

*(5) Soaking/Wetting*. Soaking or wetting, like other food processing techniques, helps to enhance product shelf life, safety, and quality [58]. A comparison of the two soaking methods revealed that soaking peeled roots was more successful in lowering cyanogen levels than soaking unpeeled roots [59]. Including the peel during the processing of cassava resulted in substantial cyanogen retention in the pulp [19]. Similarly, cassava flour mixed with water and the resulting wet flour placed in the shade for 5 hours at around 30°C to allow HCN gas to escape resulted in three- to six-fold reductions in the total cyanide level [60].

*(6) Fermentation*. Fermentation is an old method of food preservation that has gained popularity in many cultures due to its nutritional value and diverse sensory qualities [21]. It increases the nutritional value of food by the production of vitamins, vital amino acids, and antinutrient breakdown [55]. An optimized enzymatic preparation of flaxseed fermentation for 48 hours with 12.5% glucosidase and 8.9% cyanide hydratase degraded 99.3% of the cyanide concentration in the flaxseed powder [61]. Similarly, fermentation of cassava flour was reported to remove 81.5% of the cyanide content of the sample [57].

*(7) Germination*. Germination is a traditional food processing technique that is effective in lowering the cyanogenic glycoside level [22]. Li et al. [62] investigated the influence of germination on the cyanogenic glycoside content of flaxseed. Flaxseed germination considerably lowers the cyanogenic glycoside concentration. Sorghum malting and mash bioacidification are also efficient in lowering the cyanogenic glycoside level. Preheating the mash to 40°C before decantation dramatically reduces the wort's dhurrin concentration and boosts the proteolytic activity. The mash bioacidification with *L. paracasei* (ND-34) decreases dhurrin to 2.34-0.08 mg/L in white wort after fully removing it from brown wort [63].

### 3.2. Glycoalkaloids

**Figure 3:**
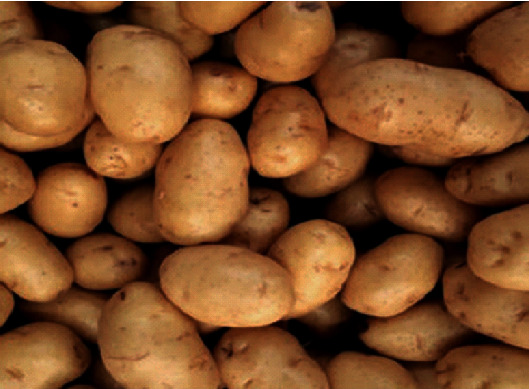
Potato tuber [69].

Glycoalkaloids (GAs) are naturally occurring toxic compounds produced by plants in the *Solanaceae* family that are associated with insect resistance [64, 65]. Potatoes (*Solanum tuberosum*), contains (*α*-solanine and *α*-chaconine), tomatoes (*Solanum lycopersicum*) (*α*-tomatine), eggplants (*Solanum melongena*) (*α*-solamargine), and aubergines (*Solanum melongena*) are all members of the *Solanaceae* family known to produce significant amounts of glycoakaloids [5]. The toxin is particularly produced in the roots, leaves, flowers, and edible parts of plants, including sprouts and skin [66–68].  Figure 3 shows the potato.

#### 3.2.1. Structure

**Figure 4:**
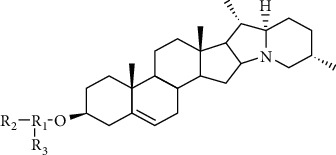
Chemical structures of *α*-solanine and *α*-chaconine.*α*-Solanine: R_1_ = D-galactose; R_2_ = D-glucose; and R_3_ = L-rhamnose. *α*-Chaconine: R_1_ = D-glucose; R_2_ = L-rhamnose; and R_3_ = L-rhamnose [72].

Glycoalkaloids are amphiphilic due to the presence of two structural components. The aglycone unit is made up of a hydrophobic cholestane skeleton with nitrogen inserted into the F-ring. The second unit is a 3-OH-attached hydrophilic carbohydrate side chain [70]. The primary *Solanum* alkaloids of both pharmacological and toxicological significance are steroidal alkamines, all of which have the cholestane C_27_ steroidal structure. They fall into one of five structural categories: (1) *α*-epiminocyclohemiketals like solanocapsine; (2) solanidine and other hexacyclic tertiary bases with attached indolizidine rings; (3) 22,26-epiminocholestanes like solacongestidine or etioline; (4) 3-aminospirostanes such as jurubidine; and (5) spirosolanes, spiroaminoketals like solasodine [70, 71]. In over 350 *Solanum* species, around 90 structurally distinct steroidal alkaloids have been identified [70].  Figure 4 shows the chemical structure of major glycoalkaloids (*α*-solanine and *α*-chaconine).

#### 3.2.2. Toxicity

The major (95%) glycoalkaloids in potato types (*Solanum tuberosum*) are *α*-solanine and *α*-chaconine, which also contain similar alkaloidal aglycone bases, solanidine. Both compounds are heat-stable, as they only decompose between 230 and 280°C [65, 73]. *α*-Chaconine, in particular, is the most poisonous of potato alkaloids in terms of total toxicity. It inhibits acetyl-cholinesterase, which causes cell disruption and organ damage, and is teratogenic in embryos; *α*-solanine on the other hand, is slightly less hazardous [65]. The *α*-solanine concentration in potatoes is usually greater than the 140 mg/kg fresh weight (FW) and has been found to cause a bitter taste and a burning feeling in the throat and mouth [74]. Furthermore, in humans, the toxic dose of total glycoalkaloid (TGA) is 2 to 5 mg/kg body weight (BW), and the lethal dose is 3 to 6 mg/kg BW [75]. The minimal acute amount of TGA is, however, around 1.0 mg/kg BW or less [76]. Their intake has been linked to diarrhea, fever, vomiting, gastrointestinal discomfort, gastroenteritis, neurological disorders, a high pulse rate, low blood pressure, and mortality in humans and farm animals [77]. As a result, the USDA has established consumption guidelines for TGAs in potatoes that should be less than 200 mg/kg FW or 1000 mg/kg DW [76].

#### 3.2.3. Glycoalkaloid Detoxification Methods

Glycoalkaloids are heat-stable molecules that remain active even after cooking the food [76, 78]. Food processing methods such as baking, cooking, and frying have no effect on the glycoalkaloide level [78, 79]. The good thing is, the majority of glycoalkaloids in potato tubers are found within the first 1 mm from the outside surface and decrease toward the tuber's center [80]. Tubers from various cultivars had an uneven distribution of *α*-chaconine and *α*-solanine, with the highest amounts around the eyes of the outer layer (periderm, cortex, and outer phloem). Before cooking, peeling the tissue 3–4 mm from the exterior removes practically all of the glycoalkaloids [81].

Boiling is claimed to remove 3.5% of the major glycoalkaloids in potatoes, but microwaving reduces amounts by roughly 15%. Temperatures above 170°C cause a significant breakdown of glycoalkaloids, whereas deep-frying at 150°C has little effect on glycoalkaloids concentrations. Heating potatoes for 10 minutes at 210°C lessens the amounts of *α*-chaconine and *α*-solanine by approximately 40% [79, 81]. A study conducted to assess the effect of industrial processing on potato granules found a significant decrease in the concentration of glycoalkaloids (*α*-chaconine and *α*-solanine) and nitrates in intermediates and finished products. Peeling (50%) and blanching (63%) resulted in the greatest reduction in glycoalkaloids, while the dried potato granules contained 14% of the initial glycoalkaloids [82].

### 3.3. Glucosinolates

**Figure 5:**
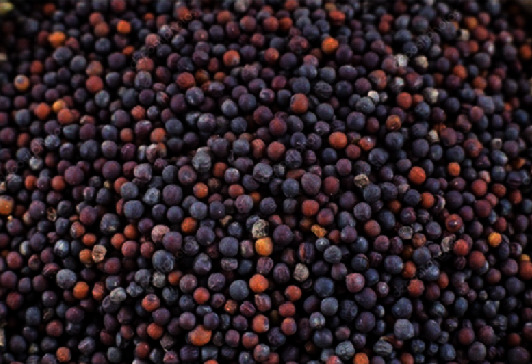
Mustard seed [88].

Glucosinolates (GSLs) are a class of chemicals found in plants such as broccoli, cauliflower, and cabbage that belong to the goitrogen family [83–85]. They have been identified in 16 different angiosperm plant species, primarily in the *Brassicaceae* family, which includes *Arabidopsis thaliana*. *Brassica* vegetables, such as broccoli (*Brassica oleracea* var. *italica*), turnip (*Brassica rapa* ssp. *rapa*), horseradish (*Armoracia rusticana*), cauliflower (*Brassica oleracea* var. *botrytis*), cabbage (*Brassica oleracea* var. *capitata*), mustard (*Brassica nigra*), and rapeseed (*Brassica napus*) [27, 83–86]. GSL levels in foliage can range from 1000 ppm in some plants to 3000 ppm in Brussels sprouts. In roots and seeds, the concentration can be significantly greater, reaching up to 60,000 ppm in mustard seed (*Brassica nigra* L.) and 30,000 ppm in horseradish root (*Armoracia rusticana*) [87].  Figure 5 shows the mustard seed.

#### 3.3.1. Structure

**Figure 6:**
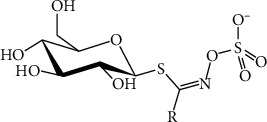
Molecular structure of glucosinolate [91].

Glucosinolates have a core structure that includes a *β*-D-thioglucose group coupled to a sulfonated aldoxime moiety and a variable chain made up of amino acids [86, 89]. After hydrolysis, GSLs generate a well-defined family of anionic natural products with the ability to form isothiocyanates (ITCs) with the common structure R-N=C=S [86]. They are derived from numerous amino acids and are classified into three categories based on their amino acid precursor: indolic GSLs from tryptophan (Trp), aliphatic GSLs from methionine (Met), and benzyl GSLs from phenylalanine (Phe) and tyrosine (Tyr) [27]. These sulfur- and nitrogen-containing compounds, as well as their metabolic byproducts, are well known for their role in plant defense against fungi, bacteria, pests, and insects [90].  Figure 6 shows the molecular structure of glucosinolates.

#### 3.3.2. Toxicity

Despite the fact that glucosinolates have a variety of health benefits, consuming vegetables and/or seeds from the *Brassicaceae* family exclusively or excessively has been linked to harmful effects [85, 92]. High levels of GSLs have been linked to a variety of harmful consequences, including an enlarged thyroid, decreased plasma thyroid hormone levels, organ abnormalities (liver and kidney), impaired growth, diminished reproductive performance, and even death [85, 93]. The enzyme myrosinase converts glucosinolates into a number of derivatives such as thiocyanates, isothiocyanates, and epithionitriles during the mastication process [85, 94]. GSLs and related chemicals have traditionally been connected to negative effects on the human body, and their ingestion has been described as causing altered thyroid function and an increased risk of numerous thyroid disorders [95]. Thyroid enlargement has been linked to long-term consumption of *Brassica* plants containing goitrin and isothiocyanates, which impede iodide uptake by the thyroid, causing an iodine shortage and, as a result, T4 inhibition. GSL antithyroid effects can cause subclinical symptoms such as impaired reproductive performance and growth or, in more extreme situations, clinically visible goiter [96, 97]. However, detailed recommendations for increasing or decreasing GLS-containing food consumption, as well as setting the maximum tolerated level are yet to be developed [84].

#### 3.3.3. Glucosinolate Detoxification Methods

Cooking is regarded as an efficient processing method to lower food's glucosinolate level [98–100]. As a result, a higher reduction of total glucosinolates after cooking has been reported in frozen *Brassica* vegetables compared to the same treatment on fresh vegetables. This could be due to blanch-freezing before boiling, which softens the vegetable [101]. Traditional boiling of cruciferous vegetables resulted in significant glucosinolate losses (90%) due to toxin leaching into the cooking water [102]. Volden et al. [103] assessed the effect of different processing methods on the glucosinolate level of cauliflower (*Brassica oleracea* L. ssp. *botrytis*). All processed cultivars showed significant (*p* < 0.05) decreases in total GLS, ranging from 18 to 22% in steamed, 30-52% in blanched, and 46-61% in boiling. Similarly, boiling and high-pressure cooking methods resulted in a 64% reduction of the total GLS content of *Brassica rapa* [104]. Cooking rutabaga, green cauliflower, and purple cauliflower resulted in a considerable decrease in total glucosinolates to 6.6, 68.9, and 69.2%, respectively [105].

Moreover, steaming decreased the total glucosinolates in broccoli after 5 and 10 min of treatment by 57.5% and 72.3%, respectively [106, 107]. Blanching of broccoli for 5 and 10 minutes, on the other hand, reduced glucosinolate levels by 62.0% and 67.7%, respectively. The maximum reduction in glucoraphanin (71.58%), the main glucosinolate in broccoli, was detected after 1 minute of boiling [106]. In the case of fermentation, bioconversion of glucosinolates into derivatives such as isothiocyanates and ascorbigen has been reported [107]. Freezing also reduced the aliphatic and total glucosinolates in frozen broccoli florets by 44.76% and 35.16%, respectively [108]. Frying red cabbage decreased total glucosinolate by 81.11% [109]. In addition, stir-frying of cabbage resulted in a 70% decrease in glucosinolate concentration [110]. Likewise, Wu et al. [109] reported a considerable reduction in the glucosinolate content of red cabbage (84.29%) after stir-frying.

### 3.4. Pyrrolizidine Alkaloids

**Figure 7:**
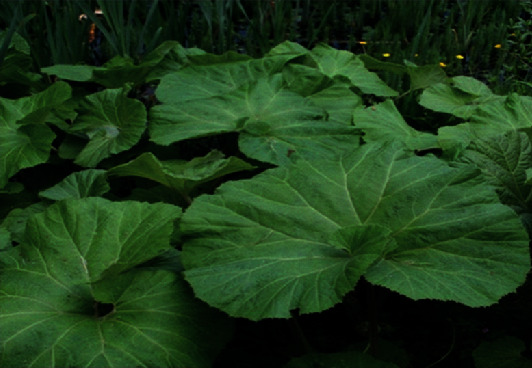
Japanese sweet coltsfoot (*Petasites japonicus*) [116].

Pyrrolizidine alkaloids (PAs) are heterocyclic chemical compounds produced by plants that are hypothesized to function as herbivore protection agents [111–113]. Plants producing pyrrolizidine alkaloids are mostly found in the *Asteraceae* (alternative name: *Compositae*) (genus *Senecio*, *Eupatoria*, and *Tussilago*), *Boraginaceae* (genus *Heliotropium*, *Symphytum*, and *Trichodesma*), and *Fabaceae* (alternate name: *Leguminosae*) families (e.g., genus *Crotalaria*) [114, 115].  Figure 7 shows *Petasites japonicus.*

#### 3.4.1. Structure

**Figure 8:**
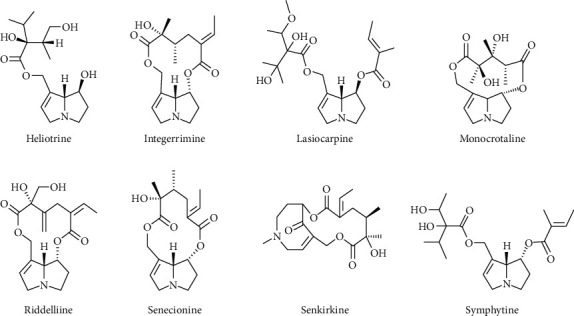
Structure of some pyrrolizidine alkaloids [121].

Pyrrolizidine alkaloids are composed of two parts, a basic amino alcohol moiety known as a necine and one or more acids (necic acids) that esterify the necines' alcohol groups. Combining known necines and necic acids can theoretically yield a wide range of pyrrolizidine alkaloids [117]. With the exception of approximately 35 otonecine alkaloids, more than 950 pyrrolizidine alkaloid structures are known to form N-oxides [115, 117]. Necines with only one hydroxyl group at C-9 have a single ester linkage with a monocarboxylic acid. Monocarboxylic acids can esterify necines with two hydroxyl groups, such as at C-9 and C-7 (7,9-necinediols), on either hydroxyl [117, 118]. The alkaloids 7-O-angeloylheliotridine and echinatine are examples of one-fold esterification. Based on chemotaxonomic considerations, around 500 distinct pyrrolizidine alkaloids and their *N*-oxides are known, and their presence is anticipated in over 6,000 plant species [113, 115, 119]. PAs are classified as monoesters, macrocyclic esters, or open-chain diesters based on the degree of esterification. At the 1,2-position, the ring system can be saturated or unsaturated, with the former being relatively nontoxic while the latter is hepatotoxic. Necine bases are classified into four types: platynecine, retronecine, heliotridine, and otonecine. There is no double bond in the base of platynecine type pyrrolizidine alkaloids, and retronecine and heliotridine are enantiomers [120].  Figure 8 depicts the molecular structure of some pyrrolizidine alkaloids.

#### 3.4.2. Toxicity

The liver is the primary target of PA toxicity, owing to the fact that bioactivation occurs mostly in this organ. Veno-occlusive disease (VOD), also referred to as hepatic sinusoidal obstruction syndrome (HSOS), is the most common clinical indication, and it is thought to be a sign of PA poisoning [113]. The symptoms associated with PA toxicity include vomiting, bloody diarrhea, and liver enlargement [122]. PA poisoning can be acute, subacute, or chronic, with each presenting a unique set of symptoms. When PAs are taken in large amounts, they can cause acute toxicity, which is characterized by hemorrhagic necrosis, hepatomegaly, and ascites; in subacute, there is a blockage of hepatic veins, which causes HSOS (primary sinusoidal damage and parenchymal cell dysfunction) [113, 115, 123, 124]. Chronic PA exposure results in necrosis, fibrosis, cirrhosis, and bile duct epithelial growth; the greatest level of toxicity is liver failure and death [113, 123, 125].

The European Commission has established maximum thresholds for the presence of PAs in a variety of commodities, including food supplements. The following values are proposed for dietary supplements including herbal components: 400 g/kg (herbal components include *Camellia sinensis* extracts) and 500 g/kg for pollen-based food supplements and pollen and pollen products [126]. In 1992, the German health authority published a “graduated plan” indicating that the maximum daily exposure to PAs from pharmaceutical products should not exceed 0.1 g for internal use and 10 g for exterior use [127].

#### 3.4.3. Pyrrolizidine Alkaloid Detoxification Methods

Soaking and boiling with peeling are indicated as effective methods for minimizing the PA content of food. PAs in the petioles and spikes of young *Petasites japonicus* decreased to less than 50% of the original amount after 1 h and 6 h of soaking and boiling, respectively [128]. Gieva et al. [111] reported the complete removal of pyrrolizidine alkaloids from essential oils of eucalyptus and lemon by hydrodistillation in their manufacturing process. There is limited scientific work on food processing methods to mitigate the PAs of plant sources [129].

### 3.5. Lectins (Phytohaemagglutinins)

**Figure 9:**
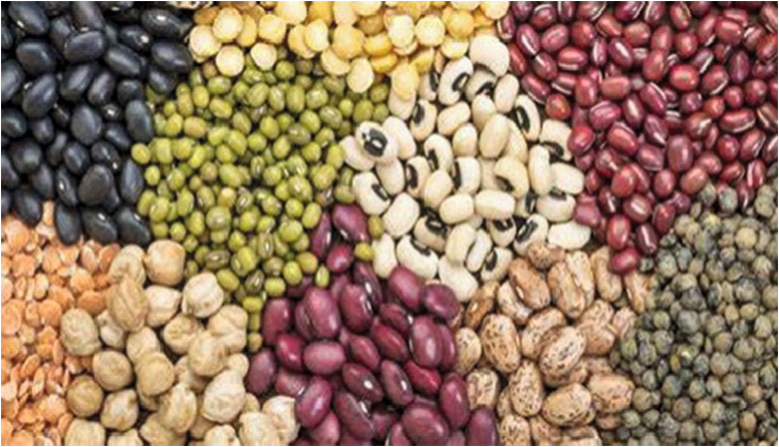
Legumes [137].

Lectins are also known as phytohaemagglutinins due to their capacity to agglutinate red blood cells [130, 131]. They are proteinaceous, poisonous chemicals found in legumes [131]. Lectins are non-immune proteins with a wide distribution in nature that identify and covalently bind to carbohydrates and glycoconjugates and are either free or attached to cell surfaces via specialized binding sites [132]. They are found in most plants, particularly seeds such as cereals, beans, wheat, quinoa, peas, kidney beans, bananas, lentils, soybeans, mushrooms, rice, and tubers such as potatoes [89, 133]. Lectins have been discovered to be more concentrated in plant seeds and to be localized in various vegetative tissues such as flowers, cereals, legumes, rhizomes, roots, leaves, bulbs, bark, fruits, phloem sap, latex, and nectar [134–136].  Figure 9 depicts different legumes known to contain lectins.

#### 3.5.1. Structure

**Figure 10:**
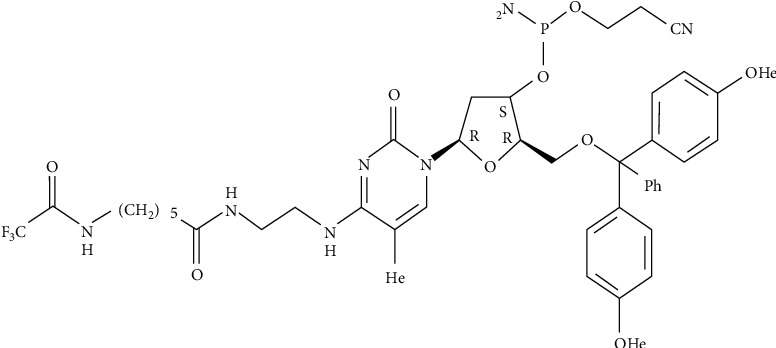
The chemical structure of a lectin found in bananas [140].

Lectins are a wide class of homologous proteins. All of the legume lectin sequences have been determined to have pairwise sequence identities of more than 35% [138]. They have at least one non-catalytic domain that binds to a particular mono-or oligosaccharide reversibly. They can aid in the recognition of glycoconjugates on the cell surface, as well as the separation and structural study of glycoproteins and oligosaccharides [130, 139]. Most legume lectins are mature protomers made up of a single polypeptide chain of about 250 amino acid residues, hence the name “one-chain legume lectins.” When two different types of polypeptide forms occur in specific legume lectins, they are referred to as two-chain legume lectins. In addition, many legume lectins are *N*-glycosylated, and the protomers of these lectins contain one or two glycan chains. Furthermore, among all plant lectins, legume lectins are the only metalloproteins with tightly bound Mn^2+^ and Ca^2+^ ions, which are responsible for their carbohydrate-binding characteristics [139].  Figure 10 depicts the chemical structure of a lectin in a banana.

#### 3.5.2. Toxicity

Upon ingestion, legume lectins bind to particular receptor sites on the intestinal epithelial cell surface, interfering with nutrient absorption across the intestinal wall in a non-specific manner [131, 141]. Once bound to the digestive tract, the lectin may alter part or all of the digestive, absorptive, protecting, and secretory functions of the entire digestive system, as well as cellular proliferation and turnover. All pulse lectins require transition metal ions and calcium binding for active confirmation loop stabilization. Thus, lectins inhibit the activity of digestive enzymes, lowering protein *in vitro* digestibility [131]. Furthermore, lectins that are not digested efficiently by digestive enzymes and have an affinity for the surface of gut epithelial cells, like those found in the *Leguminosae* family, can be toxic [142]. By breaking down the surface of the small intestine, lectins can circumvent the human defense system and spread all over the body, causing disorders (such as Crohn's disease, coeliac disease, and colitis) [132, 143].

A high lectin concentration in food causes nutritional deficits, gastrointestinal distress, immunological allergic reactions, and food poisoning [135, 144]. Lectins may also be a cause of severe inflammation and devastation of epithelial cells, as well as edema, hyperemia, and hemorrhages in lymphatic tissues [135, 145]. Lectins can induce cells to act as though they have been activated by insulin or cause the pancreas to release insulin. By providing incorrect immune system codes and promoting the proliferation of some white blood cells, lectins can also induce autoimmune disorders [132, 146, 147]. Concanavalin A (ConA), wheat germ agglutinin (WGA), phytohaemagglutinins (PHA), and the lectin from *R. pseudoacacia* were discovered to be harmful to mammalian cells *in vitro* and *in vivo* [135].

Acute toxicity of lectin is caused by the binding of lectin to the intestinal mucosa membrane or the villi of the intestinal lumen [135, 148]. The PHA agglutinin (phytohemagglutinin of *Phaseolus vulgaris*) alters the permeability of the intestinal membrane. It is characterized by diarrhea, nausea, and vomiting [135]. Chronic toxicity occurs in the presence of repeated exposures to lectins with resistance to the gastrointestinal tract of certain cellular processes generated by the binding of lectin receptors of epithelial cells and the action mechanism demonstrated by these lectins [135, 149]. PHA also acts as a mitogen in the crypt of villi cells, causing hyperplasia and growth in the small intestine [135, 150]. Long-term intake in rodent models is associated with accelerated cell turnover, weight loss, and intestinal hyperplasia. The lethal dose (LD_50_) values for various lectins have been recorded for mice (abrin, 0.02 mg/kg body weight), rats (PHA, 50 mg/kg body weight), and humans (SBA, 50 mg/kg body weight) [135].

#### 3.5.3. Lectin Detoxification Methods

Heating plant sources during the cooking process can drastically reduce the amount of lectins in them [132]. PHA and other lectins are usually heat-sensitive and can be eliminated by proper processing. Fermentation of lentils (*Lens culinaris*) for 72 hours at an optimal temperature has been reported to totally eliminate the seed's lectin content [151]. Soaking the seeds in distilled water greatly reduced their lectin content (0.11-5.18%). It also reduced the hemagglutinating activity of lectins in peas, lentils, fava beans, chickpeas, common beans, and soybeans by 3.13–4.09%, 1.41–4.33%, 0.62–5.18%, 0.11–3.51%, 0.73–2.44%, and 0.82%, respectively. Cooking was much more effective than soaking at lowering hemagglutinating activity in pulses and beans, with decreases ranging from 93.77 to 99.81% [152].

## 4. Emerging Food Processing Methods in Detoxifying Natural Food Toxicants

Various novel food-processing methods have been developed in recent years to improve both the palatability and durability of food. Moreover, these methods are widely employed in detoxifying a range of toxicants from food.  Table 1 summarizes some of the novel food processing techniques applied to detoxify naturally occurring food toxicants.

## 5. Discussion

Naturally occurring toxic phytochemicals are produced by the plants of food sources that contribute to a significant portion of the global population's diet. For instance, cassava is the major staple food in most African communities and has economic value in Africa, South America, and Southeast Asia. Over half a billion of the world's population depends on cassava as their major staple [5, 160]. According to the Food and Agriculture Organization, cassava is ranked third, after rice and corn, as the most important source of calories in the tropics [161]. Similarly, sorghum is also among the most important crops in Africa, Asia, and Latin America [19], as it is the fifth most-produced cereal grain [63]. Likewise, potatoes are used as a source of carbohydrates for hundreds of millions of people as well as a crop vital to the economies of South America, Africa, East Asia, and Central Asia [162]. Despite their advantages, they contain a significant amount of toxic secondary metabolites. The majority of societies in these low- and middle-income countries rely on these limited diet sources, thus prolonged exposure to significant amounts of toxic compounds from plants may have negative health consequences.

On the other hand, the literature portrayed positive health benefits, including anticancer, antimicrobial, anti-inflammatory, and other related health effects of glycoalkaloids, glucosinolates, lectins, and pyrrolizidine alkaloids in limited amounts. As a result, appropriate food processing methods that are effective in minimizing the toxic secondary metabolites to the safest limit should be encouraged. Various conventional food-processing methods have been pointed out by different researchers as effective means of detoxifying these toxicants. Traditional food processing methods, on the other hand, often degrade the nutritional quality of processed foods. Emerging food processing approaches have been shown to maintain nutritional quality while also being helpful in detoxifying naturally occurring phytotoxins. However, in low- and middle-income countries, they have limited applicability and accessibility. Thus, to meet the growing world food demand and ensure food security, it is recommendable to work on technology adoption and appropriate food processing techniques to minimize exposure to naturally occurring toxicants.

## 6. Conclusions

Plants serve as a major source of food for most living organisms. Some plants evolve defense mechanisms to protect themselves from predators by producing inherent chemicals known as secondary metabolites. These secondary metabolites such as cyanogenic glycosides, glucosinolates, glycoalkaloids, lectins, and pyrrolizidine alkaloids are widely found in the most important plant foods, which include cassava, flaxseed, potato, broccoli, rapeseed, chickpea, soybean, and lentil. These toxicants are commonly produced by most of the staple food sources in developing countries. They are beneficial for the plant itself but toxic to other organisms, including human beings. According to the literature, some of these toxic chemicals have health benefits and are used to protect against chronic health complications such as cancer. Inversely, short- and long-term exposure to significant amounts of these phytotoxins may end up with chronic irreversible negative health problems in important organ systems such as the immune system, kidneys, and reproductive system, and in severe cases, they can be carcinogenic and fatal. As a result, regulatory bodies in some countries have set a maximum tolerable limit for these toxicants in food and herbal products. Naturally, toxic chemicals occur in amounts much higher than the established limits. Thus, researchers and processors have introduced different traditional and emerging food processing techniques that could significantly reduce most of the toxicants in food to the safest level. Despite their ability to preserve the nutritional value of processed foods, emerging food processing methods have limited application and accessibility in middle- and low-income countries. As a consequence, much more work should be done on the implementation of emerging technologies, with additional scientific work recommended on food processing methods that are effective against these naturally occurring plant food toxicants, particularly pyrrolizidine alkaloids, with minimal processing.

## Figures and Tables

**Table 1:** Summary of some novel food processing techniques effective in detoxifying inherent plant food toxicants.

Toxins	Matrix	Processing method	Processing condition	Result	Reference
Cyanogenic glycoside and hydrogen cyanide	Cassava juice	Ultrasonic treatment	UPT (45°C, 81 W) for 10 min	Cyanogenic glycoside-24.95% Hydrogen cyanide-40.36% in cassava juice	Zhong et al. [153]
	Plum kernel	Microwave heating and hydrothermal treatment	Microwave heating (450 W for 6 min) combined with hydrothermal treatment (12 h at 45°C)	Microwave heating reduced 98.02% of the cyanogenic glycoside content of the plum kernel	Sheikh et al. [23]
	Flaxseeds	Microwave heating	Frequency- 2,450 MHz Power output- 450 W for 12 min	70.9% HCN removal	Safdar et al. [154]
		Sonication	Power- 300 W and 20 kHz frequency in a pulsed mode (10 s on and 3 s off) at 50°C for 20 min	92.1% HCN removal	Safdar et al. [154]
		Autoclaving	Autoclaving at 120°C for 20 min	62.6% HCN removal	Safdar et al. [154]
		Solvent extraction	Methanol: ammonia: water; in 95 : 5 : 5 at 500 rpm for 15 min	82.4% HCN removal	Safdar et al. [154]

Glycoalkaloids	Potato	High-pressure processing (HPP) and ultrasound treatment (US)	HPP treatment-600 MPa Duration- 3 min at 10.6°C	Reduced 95% glycoalkaloid content of rooster potatoes	Tsikrika et al. [155]

Glucosinolates	Rapeseed meal	Steam explosion	Steam pressure- 1.6 MPa Duration- 180 s	99% of glucosinolates removed	Gu et al. [156]
	Broccoli sprouts	High-pressure processing (HPP)	Pressure- 600 MPa for 3 min	85% of glucosinolates converted into health-benefiting isothiocyanates	Westphal et al. [24]
	Broccoli	Microwaving	Power- 1000 W Duration- 10 min	64.6% glucosinolate content reduced after microwaving	Hwang & Kim [106]
	Indian mustard	Microwave treatment	Frequency of 2450 MHz, power- 900 W Duration- 6 min	Glucosinolates level reduced from 20.46 *μ*M g^−1^ to 3.57 *μ*M g^−1^	Verma et al. [157]

Lectins	*Sebastiania jacobinensis* bark	Gamma irradiation	Radiation dose- 1 kGy Duration-1 hr	Significant loss of lectin activity	Vaz et al. [158]
	Chickpea Soybean Lentil Peanut	Instant controlled pressure drop	Steam pressure (up to 8 bar) Temperature- 170°C Duration- 3 min	Haemagglutinating activity of lectin is significantly reduced	Pedrosa et al. [159]

## Data Availability

The raw data supporting the conclusion of this article will be made available by the authors without undue reservation.
